# Test your knowledge and understanding

**Published:** 2014

This page is designed to help you test your own understanding of the concepts covered in this issue, and to reflect on what you have learnt. We hope that you will also discuss the questions with your colleagues and other members of the eye care team, perhaps in a journal club. To complete the activities online – and get instant feedback – please visit www.cehjournal.org

**Table:** 

**Question 1. Which of the following are known avoidable risk factors for exudative age-related macular degeneration?**	**Select one**
a.	Smoking	□
b.	Previous cataract surgery	□
c.	Genetic factors	□
d.	Malnutrition	□
**Question 2. Anti VEGF injections are INEFFECTIVE in which 1 of the following conditions?**	Select one
a	Exudative age-related macular degeneration	□
b	Diabetic macular oedema	□
c	Atrophic age-related macular degeneration	□
d	Central retinal vein occlusion	□
**Question 3. Which of the following is an important early feature of exudative age-related macular degeneration?**	Select one
a	Distorted vision	□
b	Painful eye	□
c	Loss of peripheral vision	□
d	Photophobia	□
**Question 4. Non-communicable eye diseases (NCEDs) are becoming a public health problem. Which of the following statements is FALSE?**	Select one
a	NCEDs have increased in importance for three reasons: because the prevalence of infectious eye diseases has decreased, because of changes in lifestyle, and because people are living longer.	□
b	NCEDs are lifelong and require long term, complex management.	□
c	There is a shortage of skilled eye care workers able to provide comprehensive eye care, which is essential for managing NCEDs.	□
d	The World Health Organization (WHO) advises that efforts to address NCEDs should be focused within the health sector.	□

## ANSWERS

1. **a. Many studies have confirmed that smoking is the most important modifiable risk factor. b.** Cataract surgery does not increase the risk of AMD. **c.** Genetic factors are very important in AMD; however, they are not avoidable, as we cannot change our parents! **d.** Vitamin supplements at very high doses may reduce the risk of AMD, but there is no evidence that malnutrition increases the risk.
2. **c. Atrophic AMD is currently not treatable. As there are no new blood vessels or leaking blood vessels, anti-VEGF drugs have no effect. a, b** & **d** can all be treated effectively with antiVEGF drugs. There is good evidence that treatment with anti-VEGF is better than observation or laser.
3. **a. Distortion is the most important early symptom of macular disease. If straight lines look bent or wavy, there is likely to be a macular problem. b** and **d.** AMD affects only the retina and choroid, so it is entirely painless and does not cause any symptoms of discomfort. **c.** AMD affects only the central retina, so patients experience a loss of central vision but have normal peripheral vision.
4. **d.** The WHO has emphasised the importance of multi-sectoral engagement, for example with education and agriculture, to effectively address the causes of NCEDs, such as diet and lifestyle (including smoking).

## Reflective learning

Visit **www.cehjournal.org** to complete the online ‘Time to reflect’ section.

